# Efficacy of two artemisinin-based combinations for the treatment of malaria in pregnancy in India: a randomized controlled trial

**DOI:** 10.1186/s12936-018-2393-3

**Published:** 2018-07-04

**Authors:** Anupkumar R. Anvikar, Irene Kuepfer, Vinitkumar Mishra, Jane Bruce, Tushar Arya, Deb Ranjan Mishra, Sanjib Mohanty, Rajesh Mohanty, Bina Srivastava, Suryakant Sharma, Neelima Mishra, Brian Greenwood, Daniel Chandramohan, Neena Valecha

**Affiliations:** 1 National Institute of Malaria Research, New Delhi, India; 2 London School of Hygiene and Tropical Medicine, London, UK; 3 Ispat General Hospital, Rourkela, India; 4 Tata Main Hospital, Jamshedpur, India; 5 National Institute of Malaria Research Field Unit, Rourkela, India

## Abstract

**Background:**

In India, the recommended first-line treatment for malaria in the second and third trimester of pregnancy is artesunate + sulfadoxine-pyrimethamine (AS+SP). However, data on safety and efficacy of artemisinin-based combination therapy (ACT) in pregnancy is limited. This study assessed the safety and efficacy of AS+SP and artesunate + mefloquine (AS+MQ) for treatment of *Plasmodium falciparum* in pregnancy in India.

**Methods:**

This open-label, randomized clinical trial was conducted from October 2010 to December 2013 at three sites in India (Ranchi and Jamshedpur in Jharkhand state, and Rourkela in Odisha state). Pregnant women in the second or third trimester who had *P. falciparum* mono-infection of any parasite density with or without fever were randomized to receive AS+SP or AS+MQ. Blood slides and filter paper samples for Polymerase Chain Reaction (PCR) were collected on days 0, 1, 2, 3, 14, 21, 28, 42 and 63 post treatment. Women were followed up at delivery and at day 42 postpartum.

**Findings:**

Two hundred and forty-eight women of 7064 pregnant women (3.5%) who were screened at monthly antenatal clinics had a *P. falciparum* mono-infection and were randomized to receive AS+SP (125) or AS+MQ (123) and all of these women were included in the intention to treat (ITT) analysis. The primary endpoint of an adequate clinical and parasite response (ACPR) on day 63 was not available for 9 women who were counted as treatment failure in the ITT analysis. In the ITT population, the ACPR was 121/125 (96.8%; 95% Confidence interval (CI) 92.0–99.1%) in the AS+SP group and 117/123 (95.1%; 95% CI 89.7–98.2) in the AS+MQ group. Among the 239 women (121 from the AS+SP arm and 118 from the AS+MQ arm) who completed the day 63 follow up (per protocol analysis) the ACPR was 100% in the AS+SP group and 99.2% (117/118) in the AS+MQ group. There were five serious adverse events (SAE) among pregnant women (4 in the AS+SP group and 1 in the AS+MQ group) and 13 fetal/neonatal SAEs (7 in the AS+SP group and 6 in the AS+MQ) but none of them were related to the study drugs. A higher proportion of women in the AS+MQ arm reported vomiting within 7 days post-treatment than did women in the AS+SP arm (6.9 vs. 1.6%; p = 0.001).

**Conclusion:**

Both AS+SP and AS+MQ are safe and effective for treatment of uncomplicated falciparum malaria in pregnancy in India.

*Trial registration* **CTRI** This study is registered with Clinical Trial Registry India (CTRI), number CTRI/2009/091/001055. Date of Registration 11 January 2010, http://ctri.nic.in/Clinicaltrials/pmaindet2.php?trialid=1185&EncHid=&userName=anvikar

**Electronic supplementary material:**

The online version of this article (10.1186/s12936-018-2393-3) contains supplementary material, which is available to authorized users.

## Background

Malaria continues to be a major public health challenge in India with about one million cases reported annually [1]. The burden of malaria in India varies markedly between different ecological zones, but it is estimated that about two percent of pregnant women suffer from malaria in endemic areas [2, 3]. Malaria is known to cause fetal as well as maternal complications during pregnancy [4]. Timely diagnosis and treatment of malaria in pregnancy is of utmost importance to prevent complications in this vulnerable group [5]. Until 2010, the Indian national policy for treatment of malaria in pregnancy (MiP) was chloroquine for *Plasmodium vivax* and quinine for *Plasmodium falciparum* infections in all trimesters. Since 2011, artesunate + sulfadoxine-pyrimethamine (AS+SP) has become the recommended treatment for falciparum malaria in the second and third trimesters of pregnancy [6]. Although there are reports of the emergence of resistance to SP in some parts of India [7], AS+SP was found effective for treatment of uncomplicated malaria in non-pregnant adults in India [8]. Artemisinin + mefloquine (AS+MQ) was also found to be safe and effective in non pregnant adults in India [9]. Evidence from other countries suggest that artemisinin-based combination therapy is superior to quinine-based therapy [10]. AS+SP and AS+MQ were found safe and effective in pregnant women in Malawi and Zambia, respectively [11, 12]. However, there is no data on the safety or efficacy of any form of artemisinin-based combination therapy (ACT) in malaria in pregnancy from India. The safety and efficacy of AS+SP and AS+MQ was assessed for treatment of *P. falciparum* malaria in pregnancy in two eastern states in India.

## Methods

This individually randomized, open label trial was conducted among pregnant women at three sites in India. Two sites (Ranchi and Jamshedpur) are in Jharkhand state and the third site (Rourkela) is in Odisha state. In all three sites, malaria transmission is perennial and the majority of the malaria cases are due to *P. falciparum* [13].

### Enrolment into cohort of pregnant women

After obtaining written informed consent, pregnant women were screened for malaria at monthly, public antenatal clinics (ANC) in the catchment area of the three study hospitals. At enrolment into the cohort and at each subsequent, monthly ANC visit, blood slides were collected from all women irrespective of whether they had a history of fever or not. In addition, women who had fever or a history of fever were tested with a malaria rapid diagnostic test (RDT) malaria Pf dipstick RDT (Guangzhou Wondfo Biotech Co. Ltd. China). If tested positive, they were referred to the study hospital for further assessment. All blood slides collected were examined on the same day. Women with a positive blood slide were invited to the study hospital the same day to assess eligibility. Women who fulfilled the inclusion criteria were enrolled into the trial and admitted to the study hospital for treatment.

Pregnant women of the study cohort of any parity in the 2nd or 3rd trimester who had a *P. falciparum* mono-infection were eligible for enrolment. The gestational age was determined by ultrasound. Women having any of the following conditions were excluded: (i) Hb < 7 g/dL; (ii) gestation of < 12 or > 36 weeks; (iii) age < 18 years (iv) abnormal liver or renal function tests; (v) a history of taking an anti-malarial within the past 7 days; (vi) a history of allergy to any of the study drugs; (vii) taking part in any other clinical trial of drugs or vaccines; (viii) severe malaria (based either on clinical presentation or hyperparasitaemia); (ix) evidence of severe concomitant infection; (x) known chronic disease (cardiac, haemoglobinopathy); (xi) a history of convulsions during the present illness or a history of psychiatric disorder or seizures (xii) vivax malaria or a mixed infection.

### Sample size

Initially, it was estimated that 500 pregnant women with *P. falciparum* malaria would be needed for inclusion in the trial based on the following assumptions: (1) day 63 adequate clinical and parasite response (ACPR) for AS+SP would be 85%, and that for AS+MQ it would be 95%; (2) loss to follow up would be < 20%; (3) the power of the study would be 90% to detect a 10% difference in ACPR between the treatment groups at a 95% significance level. Because of very low treatment failures in both arms and a lower than expected enrolment rate the sample size was subsequently revised to 300. Study enrolment was stopped at the end of the malaria transmission season in December 2013 when a total of 248 pregnant women were enrolled.

### Randomization

Treatment allocation envelopes were prepared by an independent statistician in permuted blocks of 12 for each site. Eligible participants were randomly assigned to either AS+SP or AS+MQ arm by selecting a sealed envelope that contained the treatment code; envelopes were labeled with unique identifier numbers and kept sequentially.

### Treatment

Enrolled women were admitted to the study hospitals for 3 days for administration of the study drugs. They received either AS+SP (artesunate 200 mg daily for 3 days and sulfadoxine 1500 mg + pyrimethamine 75 mg on day one) or AS+MQ (artesunate 200 mg + mefloquine 440 mg daily for 3 days).

### Definitions of birth outcomes

Miscarriage was defined as loss of the fetus before 28 weeks of gestation. Abortion was defined as premature expulsion of the products of conception from the uterus. Intrauterine death defined as fetal death prior to complete expulsion or extraction from the mother. Stillbirth: baby born with no signs of life at or after 28 weeks’ gestation.

### Assessments

During the first 3 days of treatment, women were assessed in hospital. Study participants were then requested to come for follow up visits on days 7, 14, 21, 28, 42 and 63 post-treatment. Women who missed follow-up visits were visited at home by the study team. A blood slide and filter paper sample were collected on day 0, 1, 2, 3, 7, 14, 21, 28, 42 and 63. Venous blood samples for haemoglobin (Hb) measurement, renal and liver function tests were collected at enrolment and on days 14 and 63. Adverse events were assessed at each scheduled and unscheduled visit and treated in a study hospital when appropriate.

After the initial 63-day follow-up period, women were seen at monthly ANC clinics and were encouraged to deliver at one of the study hospitals. They were given free transport to reach the study hospital for delivery and postnatal care.

At delivery, peripheral blood samples and placental biopsy samples were collected for all institutional deliveries. When deliveries occurred outside the facility, home visits were conducted as soon as possible after birth by a study midwife. Birth-weight, gestational age (Ballard score), and the presence of congenital abnormalities were assessed at delivery or within 7 days of birth. All participants were followed up on day 42 post-partum to assess the health status of the mother and her baby.

Rules for administration of rescue therapy with quinine were: (1) early or late treatment failure; (2) > 3 g/dL drop in haemoglobin or a haemoglobin level < 7 g/dL during treatment. The rule for stopping the trial was an unacceptable frequency of adverse events. The Data Safety and Monitoring Board (DSMB) reviewed data regularly and had the authority to recommend to the sponsor stopping the trial if they had major concerns.

### Laboratory methods

Thick and thin blood films were Giemsa-stained and examined by two microscopists for species identification. Parasite density was ascertained by counting asexual parasites against 200 white blood cells in the thick film. Parasites were counted against 500 WBCs if the count was less than 99 parasites per 200 WBCs. For determining the parasite density, a white blood cell count of 8000/μL was assumed. For discordant readings on parasite density the mean was taken if the difference was < 25%. For any other discordant results, the slides were read by a third microscopist whose findings were considered as the final result.

In cases of treatment failures, PCR genotyping was carried out to differentiate between recrudescent and new *P. falciparum* infections following WHO PCR genotyping guidelines [14]. Genotyping was done sequentially using the molecular markers *msp2*, *msp1* and *glurp*. PCR analysis was done at the National Institute for Malaria Research (NIMR) in Delhi on paired blood samples collected on day zero and on the day of failure.

### Data management

Case Report Forms (CRFs) were filled manually. GCP-monitoring, including source data verification, was done for all CRFs before data was double entered and verified in OpenClinica. Data processing and analysis was done using the statistical software package STATA Version IC12.0 (STATA TM, Stata Corp, USA).

### Statistical analyses

Baseline characteristics at enrolment, parasite clearance rates and birth outcomes were summarized and compared across treatment groups. Binominal exact method was used to calculate confidence intervals. Chi square test and *t* test were used to test the level of significance for categorical and continuous variables, respectively. The primary trial endpoint, adequate clinical and parasite response (ACPR), was defined as absence of parasite from day 3 until day 63 post-treatment. Treatment efficacy was assessed in the intention to treat (ITT) population, which included all women enrolled in the study, and in the per protocol (PP) population, which included all women who had completed treatment and the follow up on day 63. In addition, a Kaplan–Meier (KM) survival analsysis was performed to compare the probability of treatment failure in both study arms, censoring the women when they were lost to follow up. Safety analysis was performed in the ITT population.

### Ethics

The protocol was approved by Ethics Committees of NIMR, Ispat General Hospital Rourkela and the London School of Hygiene and Tropical Medicine, London, UK (LSHTM). Written informed consent was obtained from all participants. An independent Data Safety Monitoring Board consisting of an obstetrician, statistician, pharmacologist and clinician monitored the safety and progress of the study.

The study is registered with Clinical Trial Registry India (CTRI), number CTRI/2009/091/001055.

## Results

**Fig. 1:**
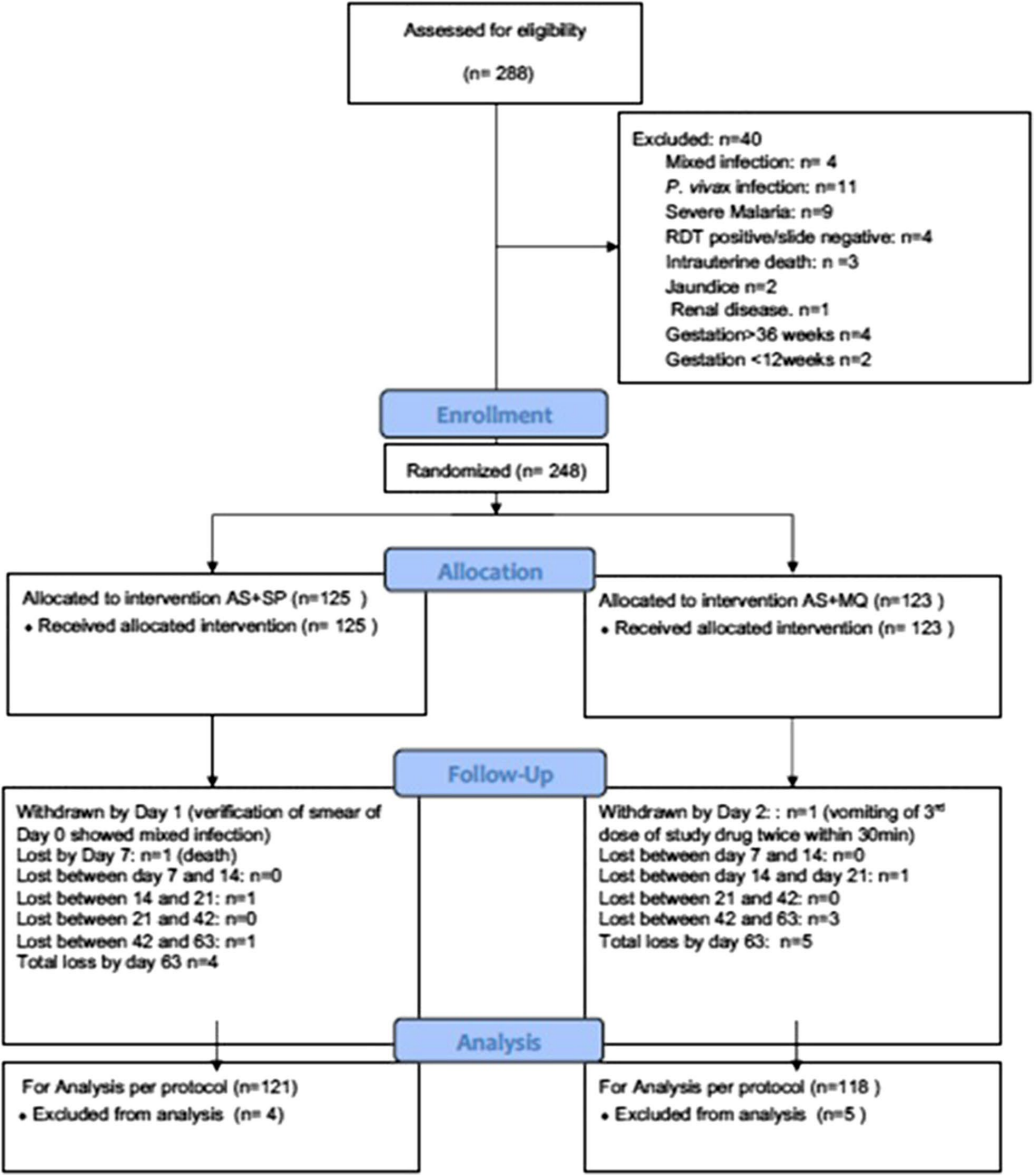
Trial consort diagram

Between October 2010 and December 2013, a total of 7064 pregnant women were screened at public ANC facilities. A total of 248 women (3.5%) were enrolled into the trial (Fig. 1). Forty women were excluded due to the presence of a mixed infection (4), a *P. vivax* infection (11), severe malaria (9), RDT positive but blood slide negative (4), intrauterine death (3), jaundice (2), renal disease (1), gestation > 36 weeks (4) and gestation < 12 weeks (2).

Mean age, gestational age, Hb concentration and parasite density at enrolment were comparable between the two study arms (Table 1). On the day of enrolment, 64.5% (160/248) of women reported a history of fever but only 24.6% (61/248) had an axillary temperature > 37.5 °C. The parasite clearance rate was similar among women in the two study arms. At enrolment, ten women had gametocytes. By day 7 all cleared gametocytes, except one subject which only cleared them by day 21. On day 3 all analysable women (246; 123 in AS+SP and 123 in AS+MQ) were free of parasites. All women enrolled in the trial were included in ITT analysis. The number of women deemed to be treatment failure due to loss to follow up were 4 in AS+SP group and 5 in the AS+MQ group. In the ITT population, the day 63 ACPR was 96.8% (121/125; (95% CI 92.0–99.1%) in the AS+SP group and 95.1% (117/123; 95% CI 89.7–98.2) in the AS+MQ group (Table 2). In the PP population (121 from the AS+SP group and 118 from the AS+MQ group) no women had a treatment failure in the AS+SP group and in this group the ACPR on day 63 was 100% (95% CI 97–100%). In the AS+MQ group, one woman had parasitaemia on day 63 (3200 parasites/µL). By PCR genotyping it was shown to be a re-infection with *P. falciparum*.. The PCR-uncorrected ACPR on day 63 in the AS + MQ group was 99.2% (117/118; 95% CI 95.4–100). Following PCR correction, it was 100% (118/118; 95% CI 96.9–100). The results of the Kaplan–Meier survival analysis showed no difference in the probability of treatment failure in both arms. The KM graph is available as Additional file 1.

**Table 1:** Baseline characteristics at enrolment, by treatment group

	AS+SP (N = 125)	AS+MQ (N = 123)	Total (N = 248)	P-value
Age (years)^a^	23.1 ± 3.9	23.6 ± 3.7	23.4 ± 3.8	0.2758
Gestation (weeks)^a^	25.5 ± 6.2	24.8 ± 5.7	25.14 ± 5.95	0.3660
Hb (g/dL)^a^	8.8 ± 1.1	9.1 ± 1.3	8.96 ± 1.2	0.1126
Asexual parasitaemia/µL^a^ [median (range)]	16,261 ± 30,494 [2800 (32–165,520)]	17,126 ± 30,705 [2220 (32–153,000)]	16,695 ± 30,540 [2400 (32–165,520)]	0.8257
Gametocyte carriage	5 (4.0)	5 (4.1)	10 (4.0)	
Gravidity				
1	57 (45.6)	52 (42.3)	109 (44.0)	0.5998
2	37 (29.6)	38 (30.9)	75 (30.2)	0.8253
3+	31 (24.8)	33 (26.8)	64 (25.8)	0.7163
Parity				
0	64 (51.2)	61 (49.6)	125 (50.4)	0.8012
1	36 (28.8)	40 (32.5)	76 (30.6)	0.5271
2+	25 (20.0)	22 (17.9)	47 (19.0)	0.6726

^a^Mean ± SD; percentages in brackets

**Table 2:** Parasitaemia from day 0 until day 63

Treatment day 0	ASSP N (%)	ASMQ N (%)
Positive blood slide	125	123
Mean parasite density/μL	16,261 ± 30,494	17,126 ± 30,705
Post treatment day		
Day 1		
Positive blood slide	24/124 (19.4)	28/123 (22.8)
Mean parasitaemia/μL	173 ± 777	1568 ± 8958
Day 2		
Positive blood slide	3/123 (2.4)	2/123 (1.6)
Mean parasitaemia/μL	0.72 ± 4.8	2.1 ± 14.8
Day 3		
Positive blood slide	0/123	0/123
Day 7		
Positive blood slide	0/123	0/122
Day 28		
Positive blood slide	0/122	0/121
Day 42		
Positive blood slide	0/122	0/121
Day 63		
Positive blood slide	0/121	1/118

There were five maternal SAEs, four in the AS+SP group and one in the AS+MQ group. In the AS+SP group, the SAEs were one maternal death, hospitalization due to oligohydramnios (2) and antepartum haemorrhage (1). The maternal death occurred on day Five due to complications of severe malaria. This woman had high parasitaemia on day 0 (205,440 parasites/µL) and, therefore, should have not been enrolled. In the AS+MQ arm there was one hospitalization due to a urinary tract infection. None of the serious adverse events were considered related to the study drugs. There were 13 fetal or neonatal SAEs, 7 in AS+SP and 6 in AS+MQ arm. The causes of fetal/neonatal SAEs in the AS+SP group were intrauterine death (4), neonatal jaundice (2), still birth (1). In the AS+MQ group they were intrauterine death (2), asphyxia (1), aspiration pneumonia (1) and still birth (2). None of these events were considered related to the study drugs.

The observed and reported adverse events during the first week following treatment are shown in Table 3. The proportion of women who reported vomiting was significantly higher in the AS+MQ group than in the AS+SP group (6.9 vs. 1.6%; p = 0.001). A substantial proportion of women reported dizziness and weakness in both arms. The mean values of haematological and biochemical parameters did not show any significant change from day 0 to day 14 in either arm (Table 4).

**Table 3:** Observed and/or reported adverse events during the first 7 days after the start of treatment

Event	ASSP (n = 125) N (%)	ASMQ (n = 123) N (%)	Total (n = 248) N (%)	p-value
Anaemia (< 11 g/dL)	18 (14.4)	17 (13.8)	35 (14.1)	0.896
Weakness	22 (17.6)	19 (15.4)	41 (16.5)	0.648
Nausea	4 (3.2)	9 (7.3)	13 (5.2)	0.146
Vomiting	2 (1.6)	15 (12.2)	17 (6.9)	0.001
Headache	7 (5.6)	3 (2.4)	10 (4.0)	0.206
Diarrhoea	2 (1.6)	6 (4.9)	8 (3.2)	0.144
Dizziness	11 (8.8)	12 (9.8)	23 (9.3)	0.795
Lower abdominal pain	8 (6.4)	4 (3.3)	12 (4.8)	0.248
Pruritus	2 (1.6)	1 (0.8)	3 (1.2)	0.571
Tinnitus	5 (4.0)	5 (4.1)	10 (4.0)	0.979

**Table 4:** Haematological and biochemical findings in women in each treatment group on day 0 and 14

Biochemical parameter	ASSP		ASMQ	
	Day 0	Day 14	Day 0	Day 14
Haemoglobin (g/dL)	8.5	8.6	8.6	8.7
RBC (million/mm^3^)	3.9	3.9	3.9	3.9
WBC (/mm^3^)	8332	8096	7712	7872
Total bilirubin (iu/L)	0.6	0.5	0.8	0.5
SGOT (iu/L)	35.84	28.12	29.2	26
SGPT (iu/L)	30.92	21.2	17.96	16.84
Blood urea (mg/dL)	23.72	20.52	24.88	20.8
Creatinine (mg/dL)	0.80	0.80	0.79	0.76
Alkaline phosphatase (iu/L)	284.64	246.6	258.96	224.8

Birth outcomes were available for 97.2% (241/248) women (121 in the AS+SP group and 120 in the AS+MQ group). The sex ratio was 843 females per 1000 males. The occurrence of low birth weight (< 2.5 kg) was higher in the AS+SP arm than in the AS+MQ arm (28.1 vs. 16.7%; p < 0.01) (Table 5). There was no statistically significant difference in miscarriages, preterm delivery or stillbirths between the two groups. Although Ballard score was used to assess gestational age, it is not presented in the manuscript as the authors were not sufficiently confident about the accuracy of the SGA diagnosis. No congenital malformations were observed. A placental biopsy sample was available for 75% (181/241) women; none showed active placental malaria defined by the presence of parasites. At delivery, one woman of each treatment group was found blood slide positive. Both deliveries took place 1, and 2 months, respectively after the day 63 follow up in AS+SP and AS+MQ arms. The two women were treated according to national policy (AS+SP). No evidence of congenital malaria was found.

**Table 5:** Birth outcome in the AS+SP and AS+MQ groups

Birth outcomes available	ASSP n (%) 121	ASMQ n (%) 120	Total n (%) 241	p-value
Mean birth weight ± SD	2569.7 ± 471.5	2684.5 ± 407.9	2627.8 ± 443.2	0.048
Low birth weight (< 2.5 kg) including pre-term deliveries	34 (28.1)	20 (16.7)	54 (22.4)	0.027
Low birth weight (< 2.5 kg) excluding pre-term deliveries	19 (15.7)	18 (15.0)	37 (15.4)	0.081
Term delivery	97 (80.2)	99 (82.5)	196 (81.3)	0.312
Pre-term delivery	22 (18.2)	18 (15)	40 (16.6)	0.403
Miscarriage	1 (0.8)	1 (0.8)	2 (0.8)	0.991
Stillbirth	1 (0.8)	3 (2.5)	4 (1.7)	0.317

## Discussion

This is the first trial of ACT in pregnant women in India to evaluate the efficacy and safety of AS+SP and AS+MQ. The incidence of malaria in the cohort of pregnant women screened at monthly intervals was 4.1% (288/7064). Due to these low patient numbers it was not possible to reach the original targeted sample size of 500 women. However, a clear result was obtained despite the reduced sample size.

Results from this study suggests that AS+SP and AS+MQ are both efficacious for the treatment of MiP in India. The day 63 ACPR and PCR-corrected ACPR were 100% in both groups in the PP population and > 95% in the ITT population. Reasons for the high efficacy observed could be that very few people in the study area had been exposed to any ACT at the time the trial was conducted, and that the parasite density was relatively low (mean parasitaemia 16,695/µL) at recruitment. The low parasite density is partly due to the fact that the majority of the trial participants was detected by active screening and did not have clinical symptoms at enrollment; on the day of enrollment only 24.6% of women had fever or a history of clinical symptoms.

The efficacy of AS+MQ and AS+SP observed in this study is consistent with that reported from India in non-pregnant adult populations. The day 63 ACPR of AS+MQ was 100% in 2005–2007 [15] and 98.3% (95% CI 90.9–99.9) in 2008 [9]. In another study carried out in 2009–2010, the day 42 PCR corrected ACPR of AS+MQ was 100% (95% CI 92.7–100) and that of AS+SP was 90.6% (95% CI 89.2–90.9) [16]. However, a high prevalence of triple (55.5%) and quadruple (23.2%) mutation in *dhps* gene was observed [7] in the north eastern states, and recent in vivo studies have shown that the efficacy of AS + SP is declining in these states [7, 17]. The day 42 PCR corrected ACPR of AS+SP ranged from 76.6 to 82.3% across sites in the north-eastern region [7]. Thus, AS+SP needs to be replaced with other artemisinin-based combinations in this region. In a multi-country trial in pregnant women, the day 63 PCR corrected ACPR was 94.8% (95% CI 93–96.1) for artemether-lumefantrine (AL), 99.2% (95% CI 98.2–99.6) for dihydroartemisinin-piperaquine (DHAPQ), 98.5% (95% CI 97.3–99.2) for artesunate-amodiaquine (AS+AQ) and 96.8% (95% CI 95.2–97.9) for AS+MQ [18]. A review article analysed the results from 48 efficacy studies. They suggest that artemisinin-based combinations are superior to quinine-based therapy [10]. Studies carried out in India in non-pregnant adults and children have shown a high efficacy of AL [19], AS+AQ [20], AS+MQ and DHAPQ [15]. These artemisinin-based combinations are potential options to replace AS+SP in India, but there is a need to evaluate them in pregnant women in India.

In this study, no serious adverse events related to AS+SP or AS+MQ were reported. However, as the sample size was just 248 subjects, the safety findings from this study need to be interpreted with caution. The incidence of vomiting was significantly higher in women who received AS+MQ than in those who received AS+SP which confirms the finding of a high incidence of vomiting when MQ was used for intermittent preventive treatment in pregnant women [21]. The PREGACT study also reported a high frequency of vomiting in AS+MQ group [18].

The observed prevalence of low birth weight (22.4%), pre-term delivery (16.6%) and stillbirth (1.7%) was comparable to the results of a study carried out in Jharkhand state in 2009 [2], where the prevalence of low birth weight, pre-term delivery and stillbirth was reported in women with/without placental parasitaemia at 26.7%/20.9%, 13.3%/5.6% and 11.8%/4.1%, respectively. There was a higher proportion of low birth weight, preterm delivery and miscarriage in AS+SP arm compared to AS+MQ arm. Although the risk of low birth weight was significantly higher in the AS+SP arm, this finding needs to be interpreted with caution because of the small sample size and of the multiple comparisons that were done.

## Conclusion

Both AS+SP and AS+MQ are equally effective for the treatment of falciparum malaria in second and third trimester pregnancies in the central region of India. AS+SP was tolerated better than AS+MQ but it appears that AS+MQ may be slightly more effective than AS+SP at reducing adverse birth outcomes. Given that resistance to SP has emerged and is spreading in India, the risk benefit ratio of other artemisinin-based combinations (such as DHAPQ and AL) needs to be evaluated in pregnant women in India.

## Additional file

**Additional file 1.** Kaplan–Meier failure estimates.
