# Supplementary figures and images for: Efficacy of two artemisinin-based combinations for the treatment of malaria in pregnancy in India: a randomized controlled trial
Source: Malar J. 2018 Jul 4;17:246. doi: 10.1186/s12936-018-2393-3 (PMC6030775; doi:10.1186/s12936-018-2393-3)

# Kaplan-Meier failure estimates

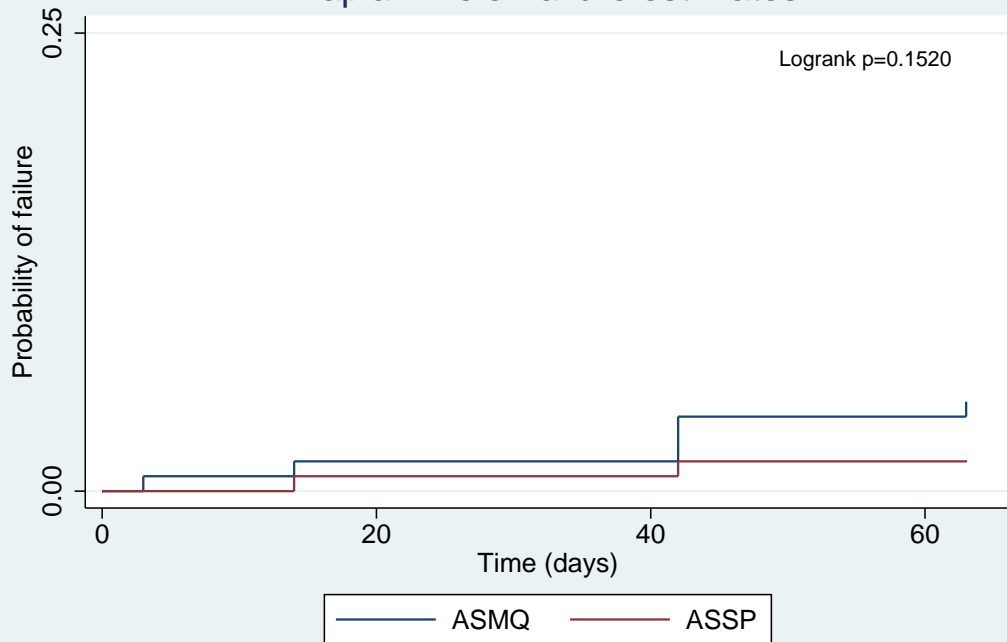

Supplement: Supplementary file 1 — Additional file 1. Kaplan–Meier failure estimates. [file 12936_2018_2393_MOESM1_ESM.pdf]
